# Seasonal variation in Ménière disease: a systematic review and meta-analysis

**DOI:** 10.3389/fneur.2026.1876181

**Published:** 2026-07-02

**Authors:** Xuanyu Shi, Linlin Wang, Xingqian Shen, Ziying Xu, Yangming Leng, Bo Liu

**Affiliations:** 1 Department of Otorhinolaryngology-Head and Neck Surgery, ENT Institute, Union Hospital, Tongji Medical College, Huazhong University of Science and Technology, Wuhan, China; 2 Hubei Province Clinical Center for Deafness and Vertigo, Wuhan, China

**Keywords:** epidemiology, Ménière disease, meta-analysis, season, systematic review

## Abstract

**Background:**

The relationship between seasonal variation and Ménière disease (MD) has not been fully elucidated. This meta-analysis aimed to investigate the association between seasonal patterns and the occurrence of MD.

**Methods:**

We systematically searched relevant observational studies in PubMed, Embase, Web of Science, the Cochrane Library, and major Chinese databases from inception to July 2025. The subjects included in the study were patients with MD. Seasonal occurrence of MD was the primary outcome. Study quality was assessed using the Newcastle-Ottawa Scale (NOS) and the Joanna Briggs Institute (JBI) critical appraisal scale. Pooled odds ratios (ORs) with 95% confidence intervals (CIs) were calculated using Stata 18.0, and heterogeneity was assessed using the *I*^2^ statistic.

**Results:**

A total of five observational studies involving 1,001,636 participants were included in the meta-analysis. With summer as the reference, ORs for spring, winter and autumn were 1.03 (95% CI 1.02–1.04), 0.92 (95% CI 0.91–0.93) and 1.04 (95% CI 0.85–1.26), respectively. Subgroup and sensitivity analysis confirmed the robustness of the findings, showing that the overall estimate was not fully driven by one single study.

**Conclusion:**

Our meta-analysis suggests that the occurrence of MD may exhibit seasonal variation across published studies, with seasonal fluctuations characterized by slightly higher occurrence in spring and declining occurrence in winter. The finding may inform patient education and disease management strategies and provide a potential role of environmental factors in the pathophysiology of MD. However, given the limited number of included studies and their geographic concentration in European and Asian populations, further high-quality studies conducted across diverse geographic regions are needed to provide more solid evidence.

**Systematic review registration:**

PROSPERO, (CRD420261348182).

## 1 Introduction

Ménière disease (MD) is a chronic inner ear disorder characterized by recurrent vertigo and cochlear symptoms, such as low to medium-frequency sensorineural hearing loss, tinnitus and aural fullness (1). It has a prevalence ranging from 3 to 513 per 100,000 individuals and represents a significant economic challenge, with annual costs in the UK estimated at £541.30–608.70 million (2, 3). Although the etiology of MD remains controversial, it is widely recognized as a multifactorial disorder. The histopathological hallmark of MD is endolymphatic hydrops (ELH), which may result from complex interplay between intrinsic and extrinsic factors (4, 5). Intrinsic factors including genetic inheritance and anatomical variations of the temporal bone remain relatively constant, whereas extrinsic factors such as viral infections (6), allergy, autoimmunity (7–9), vascular compromise etc. are potentially modifiable and may follow seasonal variations. Consequently, identifying modifiable triggers represents a crucial strategy for the preventative management of MD (2).

Meteorological factors such as atmospheric pressure, temperature and humidity have been presumed to be associated with vertigo or MD (10, 11). These meteorological parameters are intrinsically linked to seasonal changes. Consequently, it is hypothesized that the occurrence of MD may exhibit a temporal pattern corresponding to specific seasons. However, clinical studies regarding seasonal variation of MD remain inconsistent. Some studies have suggested differences in occurrence of MD, particularly between summer and winter. For example, Chen et al. reported a significantly higher occurrence of MD in summer compared to in winter in Taiwan (11). Moreover, a nationwide retrospective study from Korea found a lower risk of MD in winter than in summer, with higher risks observed in spring and autumn (12). In contrast, other studies have reported no clear seasonal pattern. A single-center prospective cohort study conducted in Germany found no significant difference in the occurrence of MD throughout the year (10). Furthermore, a multi-center cross-sectional study based on the Disease Analyzer database in Germany, including patients from 116 otolaryngology practices, reported no seasonal variations in occurrence of MD (13).

Therefore, we conducted this systematic review and meta-analysis to evaluate the impact of seasonality on the occurrence of MD. To our knowledge, this is the first meta-analysis to comprehensively examine the seasonal variation in MD. The findings may enhance our understanding of the etiology of MD and improve the preventative and therapeutic strategies of recurrent MD attacks.

## 2 Methods

### 2.1 Study design

This systematic review was conducted in accordance with the Preferred Reporting Items for Systematic Reviews and Meta-Analyses (PRISMA) statement (14) (Supplementary material 2) and the Meta-analysis of Observational Studies in Epidemiology (MOOSE) guidelines. The study has been duly registered with the International Prospective Register of Systematic Reviews (PROSPERO) and registration number is CRD420261348182.

### 2.2 Search strategy

We searched PubMed, The Cochrane Library, Embase, Web of Science, SinoMed, WanFANG, and CNKI from the establishment of the database through July 2025 for cohort studies, case–control studies, and cross-sectional studies dealing with the seasonal variation of MD. We utilized the combination of database-specific subject headings (such as Medical Subject Headings [MeSH] terms) and free-text terms to identify potentially eligible studies. General search terms such as “Meniere Disease,” “Endolymphatic Hydrops,” “Seasons,” “Climate,” “Weather,” “Air,” “Air Pressure,” “Humidity” were used. For full search strategies please see Supplementary material 1. Additionally, all titles and abstracts were evaluated, and we manually reviewed references from primary and review articles to identify any additional relevant studies (Supplementary material 1). The study protocol was structured according to the Population, Intervention, Comparison, and Outcome (PICO) framework.

### 2.3 Study inclusion

Observational studies, including cohort studies, case–control studies, and cross-sectional studies, published as full-length articles in peer-reviewed journals were eligible for inclusion. Eligible participants were required to have a clinical diagnosis of MD based on established diagnostic criteria such as the diagnostic scale of the Equilibrium Committee of the American Academy of Otolaryngology-Head and Neck Surgery (AAO-HNS) (2) or the International Classification of Diseases (ICD) diagnostic code for MD (H81.0) (15) and so on. The primary outcome was the seasonal occurrence of MD.

Two trained authors (Shi and Wang) independently screened all titles and abstracts according to predefined inclusion and exclusion criteria. Full-text articles were retrieved and further assessed when eligibility could not be determined from the abstract alone. Any disagreements were resolved through discussion and consensus with a third independent reviewer (Liu). Studies were excluded if they were reviews, case reports, animal studies, conference abstracts, theses, duplicate publications, or clearly irrelevant to the research question.

### 2.4 Risk of bias assessment

Risk of bias was assessed in accordance with the methodological principles recommended by the Cochrane Collaboration. Cohort study was evaluated using the Newcastle–Ottawa Scale (NOS) (16), while cross-sectional studies were assessed using the Joanna Briggs Institute (JBI) critical appraisal scale. This NOS assesses methodological quality across three domains: selection, comparability and exposure or outcome categories, with scores ranging from 0 to 9 points, where higher scores indicate superior quality. The JBI critical appraisal checklist consists of eight items, including sample inclusion criteria, study subject and setting description, exposure measurement, condition measurement objectivity, confounding factor identification, confounding factor mitigation strategies, outcome measurement and statistical analysis. Each item was rated as “yes,” “no” or “unclear,” and total scores ranged from 0 to 8 based on the number of “yes” responses. A score of “yes” for less than 5 times was considered low methodological quality (17) (Table 1).

**Table 1:** The characteristics of included studies assessing the seasonal variation of MD.

Number	First author publication year	Country	Study type	Data source	Diagnostic criteria	Study period	Sample size	Quality assessment
1	Yuan (2021) (20)	China	Cross-sectional	The Vertigo Clinic of the First Hospital of Shanxi Medical University	Chinese guideline of diagnosis and treatment of Meniere’s disease	2019.1–2019.12	79	5^b^
2	Ogita (2010) (21)	Japan	Cross-sectional	Emergently hospitalized in the Otolaryngology Department of Kyoto University Hospital with vertigo	Japan Society for Equilibrium Research Guidelines	2000.11–2009.8	27	6^b^
3	Gürkov (2016) (10)	Munich, Germany	Prospective cohort	A tertiary care vertigo and balance clinic in Munich, Germany	AAO-HNS guidelines	2004.10–2009.7	577	6^a^
4	Chen (2023) (11)	Taiwan, China	Cross-sectional	Neurotological Clinic of the Catholic Cardinal Tien Hospital	AAO-HNS guidelines	2015–2017	953	6^b^
5	Jeong (2024) (12)	Korean	Cross-sectional	The National Health Insurance Service of Korea	ICD-10 code H81.0	2008–2020	1,000,000	8^b^

^a^Cohort study was evaluated using the Newcastle–Ottawa Scale. ^b^Cross-sectional studies were assessed using the Joanna Briggs Institute critical appraisal scale.

### 2.5 Data extraction and season definition

Two researchers independently conducted the data extraction using a standardized data extraction form. Extracted data were cross-checked to ensure data accuracy and completeness, and any discrepancies were resolved through discussion and consensus. The extracted data in each study were as follows: authors, country, publication year, population source, types of study to be included, sample size, disease diagnostic criteria, outcome data (Table 1). As all included studies were conducted in the Northern Hemisphere, when calculating seasonal occurrence of MD, seasons were defined as follows: Spring (March, April and May), Summer (June, July, and August), Autumn (September, October, and November), and Winter (December, January, and February).

### 2.6 Statistical methods

In this study, the primary outcome was defined as the occurrence of MD. Statistical analyses were performed using Stata 18.0 software. Two authors independently extracted and analyzed categorical variable from the included studies. For categorical outcomes, odds ratios (ORs) with corresponding 95% confidence intervals (CIs) were extracted or calculated as effect measures.

Heterogeneity in pooled statistical data was comprehensively assessed by using *I*^2^ statistics and Q-test. Heterogeneity was characterized as *I*^2^ > 50% or *p* ≤ 0.1, indicating significant variability among the studies. When *I*^2^ ≤ 50% and *p* > 0.1, no significant heterogeneity was considered present, and the fixed-effect model (Inverse Variance) was employed. Conversely, when *I*^2^ > 50% or *p* ≤ 0.1, significant heterogeneity was identified, and the random-effects model (I–V heterogeneity) was utilized. Potential sources of heterogeneity were explored through subgroup analyses. Sensitivity analyses were conducted to assess the robustness of the pooled estimates. Publication bias was assessed using Egger’s regression test and funnel plots.

## 3 Results

### 3.1 Literature search and study selection/study characteristics

**Figure 1:**
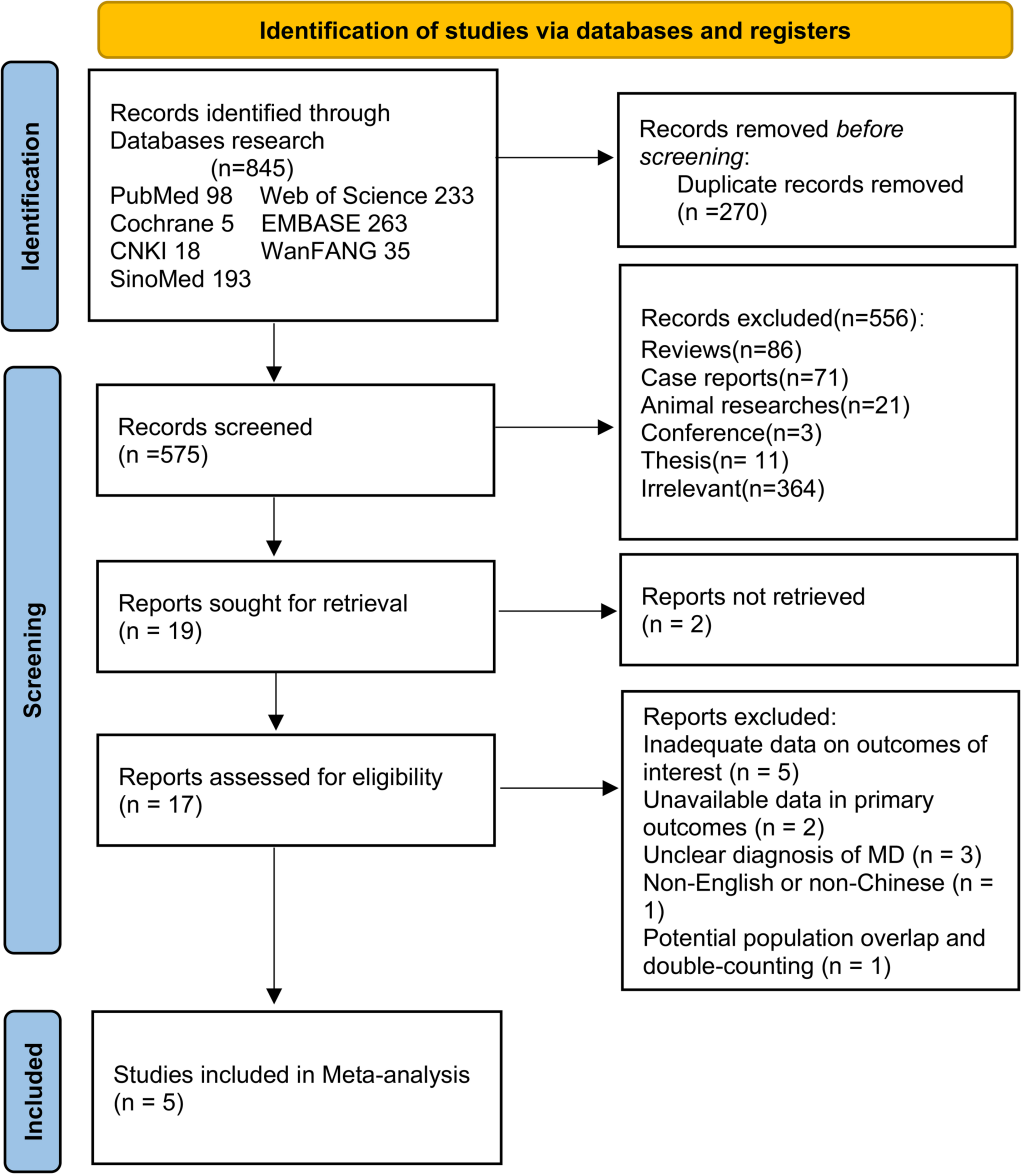
Flowchart of search results and study retrieval.

The initial search identified 845 records. After removal of duplicates and screening titles and abstracts, 19 full-text literatures were assessed for eligibility. Following a full-text review, 14 studies were excluded and 6 articles were initially considered for the meta-analysis. However, two studies utilized data from the Korea National Health Insurance Service (NHIS) database (12) and the National Health Insurance Service–National Sample Cohort (NHIS-NSC) (18). Given that the NHIS-NSC was derived from the NHIS and that the study period of Lee et al. (18) was encompassed within that of Jeong et al. (12), Lee et al. (18) was excluded from the meta-analysis to avoid potential population overlap and double-counting (19).Finally, 5 studies were included in the meta-analysis. All studies were observational in nature, of which 1 was a prospective cohort study and 4 were cross-sectional studies (Figure 1) (10–12, 20, 21).

Geographically, the studies were conducted in China, South Korea, Germany and Japan. Four studies were based on clinical data from university-affiliated hospital (10, 11, 20, 21), and one study utilized data from the NHIS database (12) (Table 1).

### 3.2 Meta-analysis

**Figure 2:**
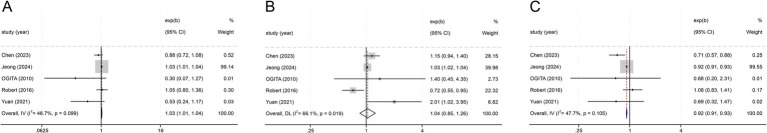
Forest plot of seasonal odds ratio (OR) for MD occurrence. Results are obtained as the OR and the accompanying 95% confidence interval. **(A)** Spring versus summer. **(B)** Autumn versus summer. **(C)** Winter versus summer.

Regarding summer as a reference, the occurrence of MD was significantly higher in spring (OR = 1.03, 95% CI: 1.01–1.04, *p* < 0.001) (Figure 2A). No statistically significant difference was observed in autumn (OR = 1.04, 95% CI: 0.85–1.26, *p* = 0.726) (Figure 2B). Conversely, the occurrence was significantly lower in winter (OR = 0.92, 95% CI = 0.91–0.93, *p* < 0.001) (Figure 2C).

### 3.3 Subgroup analysis

**Figure 3:**
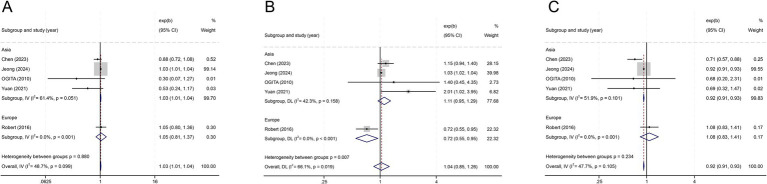
The subgroup meta-analysis based on the geographic regions. Results are obtained as the OR and the accompanying 95% confidence interval. **(A)** Spring versus summer. **(B)** Autumn versus summer. **(C)** Winter versus summer.

Subgroup analysis aims to identify the potential sources of heterogeneity and evaluate the robustness of observed outcomes. Given that the prevalence and occurrence of MD vary according to the ethnic and geographic background (3), we conducted subgroup analysis based on the geographic regions. In the Europe subgroup, the occurrence of MD was significantly lower in autumn compared with summer (OR = 0.72, 95% CI = 0.55–0.95, *p* < 0.05). However, the Asian subgroup revealed no statistical difference in occurrence of MD in autumn comparison (OR = 1.11, 95% CI = 0.95–1.29, *p* > 0.05). The test for subgroup differences was statistically significant (*p* = 0.007) (Figure 3). However, no statistically significant subgroup differences were observed in the winter comparisons (*p* > 0.05) and in spring comparisons (*p* > 0.05) (Figure 3). Since the European subgroup included only one study, these regional subgroup findings should be interpreted cautiously.

**Figure 4:**
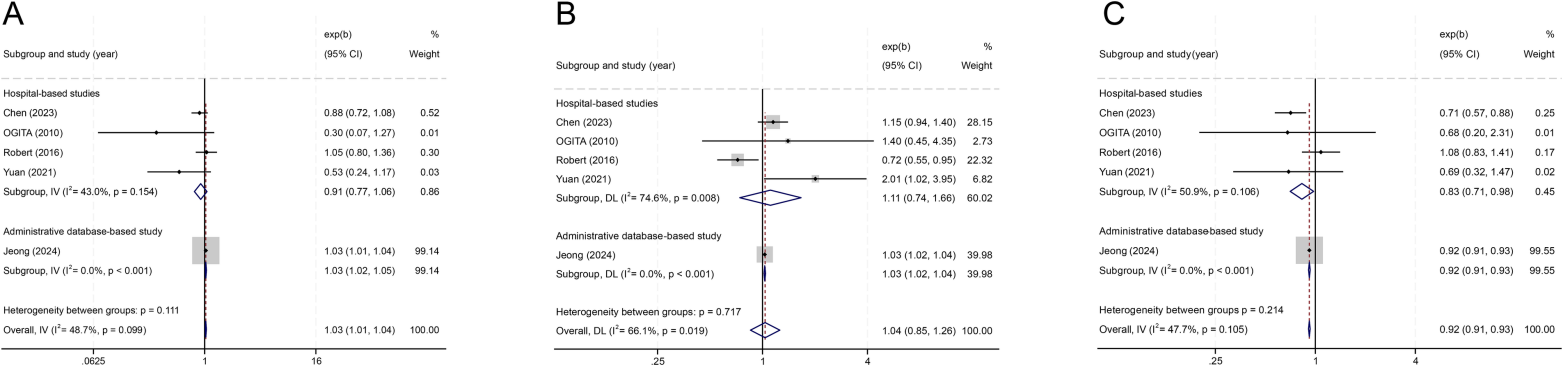
The subgroup meta-analysis based on study data source. Results are obtained as the OR and the accompanying 95% confidence interval. **(A)** Spring versus summer. **(B)** Autumn versus summer. **(C)** Winter versus summer.

Considering that different data sources may reflect different outcome types related to MD occurrence, we performed subgroup analysis by study data source. Jeong et al. (12) was classified as an administrative database-based study, whereas the remaining studies were classified as hospital-based studies. Subgroup analysis revealed results that were not entirely consistent with the primary analysis. Compared with summer, the statistically significant difference observed in spring in the primary analysis was not replicated in the hospital-based studies (OR = 0.91, 95% CI = 0.77–1.06, *p* > 0.05) (Figure 4A). And the autumn comparison revealed no statistical difference (OR = 1.11, 95% CI = 0.74–1.66, *p* > 0.05) (Figure 4B). In contrast, the occurrence of MD remained significantly lower in winter in hospital-based studies (OR = 0.83, 95% CI = 0.71–0.98, *p* < 0.05) and the administrative database-based study (OR = 0.92, 95% CI = 0.91–0.93, *p* < 0.05) (Figure 4C). No statistically significant subgroup differences by data source were detected in any comparison (*p* > 0.05).

### 3.4 Sensitivity analysis

**Figure 5:**
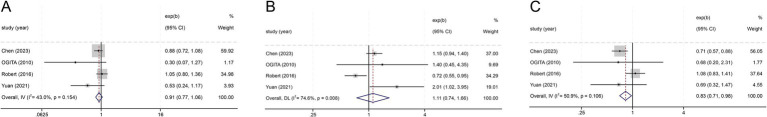
Sensitivity analysis for the occurrence of MD for different seasons compared with summer. **(A)** Spring versus summer. **(B)** Autumn versus summer. **(C)** Winter versus summer.

Subsequently, we conducted a sensitivity analysis by excluding study derived from administrative datasets to evaluate the stability of the pooled estimates (Figure 5). The pooled estimates showed no substantial variation in the winter comparison, which indicates the robustness of the results.

### 3.5 Publication bias

**Figure 6:**
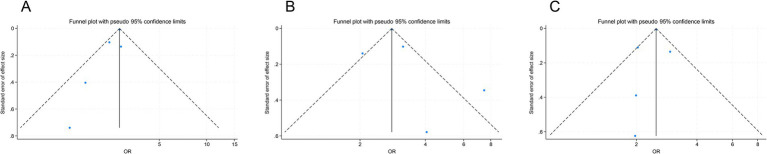
Funnel plots for the occurrence of MD for different seasons compared with summer. The abscissa is the OR value and the ordinate is the standard error. **(A)** Spring versus summer. **(B)** Autumn versus summer. **(C)** Winter versus summer.

The funnel plots for the occurrence of MD in spring, autumn, and winter were symmetrical, and mainly concentrated in the middle part. Most of the data corresponded to points within 95% CI (see Figure 6). Given the limited number of included studies, these plots were interpreted descriptively.

## 4 Discussion

This systematic review and meta-analysis synthesized findings from 5 studies encompassing 1,001,636 participants to assess the seasonal variation of MD. Our results showed that, compared with in summer, the occurrence of MD was significantly lower in winter and significantly higher in spring. Although the observed effect sizes were modest (ORs ranging from 0.92 to 1.04), such magnitudes are consistent with those reported in large-scale seasonal epidemiological meta-analyses of other chronic conditions (22). Moreover, subgroup and sensitivity analyses further confirmed the consistency and robustness of these findings. Notably, the lower occurrence of MD observed in winter appears to be consistent across sensitivity and subgroup analyses, supporting the reliability of this finding.

However, these findings should be interpreted with caution. First, the included studies were based on different study designs and outcome types. For the purpose of pooled analysis, we treated the various study outcomes as the occurrence of MD. However, administrative database studies (e.g., NHIS) mainly reflect diagnostic coding or healthcare utilization, whereas hospital-based studies may capture acute presentations rather than true incident cases. This may introduce conceptual heterogeneity in the pooled analysis. Second, the spring and winter comparisons were largely driven by Jeong et al. (12), a large Korean administrative database study that contributed the majority of participants. This may have led to relatively narrow confidence intervals to some extent. Furthermore, the occurrence of MD in the study by Jeong et al. (12) was based on health insurance claim codes, which may not reflect the actual time of MD onset. Healthcare-seeking behavior, access to medical services, and seasonal fluctuations in outpatient visit volumes could also have influenced the database records. For instance, lower diagnosis rates during winter might be partly attributable to a reduction in outpatient visits rather than a genuine decline in MD incidence. Therefore, although some comparisons reached statistical significance, the clinical significance of these findings should still be interpreted cautiously.

### 4.1 The association between meteorological (co-)factors and MD

The mechanisms underlying the seasonal variation of MD are complex. Meteorological factors, including atmospheric pressure, temperature, humidity, and special meteorological events such as typhoons, may be involved in the observed seasonal pattern.

Previous studies have demonstrated the relationship between MD and atmospheric pressure (10, 11, 20, 23, 24), humidity and temperature. The study by Schmidt et al. (24) found that lower atmospheric pressure was significantly associated with higher odds of occurrence of MD. However, the mechanisms by which occurrence of MD is influenced by low atmospheric pressure remain unclear. One hypothesis suggests that decreased atmospheric pressure may lead to reduced pressure in the middle ear cavity via the ventilation function of the eustachian tube (25). The middle ear pressure then equalizes with peri-lymphatic pressure through the round window membrane, and the decreased peri-lymphatic pressure may result in compensatory expansion of the endolymphatic compartment, i.e., ELH, potentially triggering occurrence of MD (26, 27). The efficacy of local positive pressure therapy on MD has lent further credence to this hypothesis (28, 29).

Temperature has also been reported to be associated with MD occurrence, as demonstrated by studies conducted by Choi et al. (23) and Chen et al. (11). One possible explanation for this relationship is that temperature is highly negatively correlated with the atmospheric pressure. Typhoon, a special meteorological event during the summer season in northwest Pacific region, may also be relevant due to the acute reduction in atmospheric pressure associated with these events. Furthermore, the duration of vertiginous episodes is longer during typhoon periods than during non-typhoon periods (11).

Humidity may also be associated with the occurrence of MD. It has been reported that a high relative humidity (above 90%) was associated with a 1.26-fold higher occurrence of MD (24). In addition, humidity usually reaches its peak during the summer, which may explain the seasonal variations observed in MD (21, 23). High humidity was independently associated with tinnitus, hearing loss, and increased odds of MD attacks (24). The presence of more humid air, being less dense and capable of absorbing more sound, may contribute to the observed reduction in hearing on particularly humid days.

Air pollution may also be involved in the seasonal variation of MD. As the concentration of and exposure to air pollutants are influenced by meteorological factors, such as atmospheric pressure, sunshine duration, and temperature (30), air pollutants should be analyzed as meteorological co-factors. Previous studies have indicated that prolonged exposure to air pollutants, particularly CO and O3, can increase the occurrence of MD (23). CO exerts its detrimental effects on the inner ear primarily through hypoxia and neurotoxicity (31). Similarly, O3 is a known risk factor for cardiovascular diseases (32). This systemic microvascular impairment might lead to ischemic damage of the inner ear, thus precipitating ELH and occurrence of MD.

In addition, physical activity and fatigue may increase during spring and summer compared with winter, which could be associated with MD symptoms, particularly among males and younger patients (12).

Taken together, meteorological factors and their interactions may contribute to the observed seasonal variation in MD occurrence. However, these hypotheses were derived primarily from previous studies and were not directly examined in this meta-analysis. Further studies are needed to clarify the underlying mechanisms and the relative contributions of individual meteorological co-factors.

### 4.2 The association between meteorological (co-)factors and MD comorbidities

It is well established that MD is a multifactorial disease and a variety of comorbidities may contribute to its development, such as migraine, allergy, autoimmune disease, viral infection, etc. The association between MD and migraine has been well documented, and it has been speculated that MD and vestibular migraine may represent a different spectrum of the same disease (33). Several previous studies conducted in Norway have reported that patients had more migraine attacks during the polar light season, especially for those migraineurs with aura, possibly due to prolonged light exposure. Additionally, weather changes have also been reported as significant triggering factors for migraine, which may correspond to the higher occurrence of MD observed in spring and summer in our meta-analysis (34–36). Vitamin D has a known immunoregulatory function. The serum level of vitamin D is typically lowest in late winter and early spring, and recent studies have reported an association between MD and lower serum vitamin D levels (37, 38). This may represent a possible explanation for seasonal changes in MD occurrence, although the relationship remains uncertain. Although the causal relationship between MD and allergy remains undetermined, MD patients tend to have a high risk of comorbid allergies or an allergy history, including allergic rhinitis, allergic asthma etc., which are significantly impacted by pollen season (39). Autoimmune mechanisms also play a significant role in MD. Several autoimmune diseases, rheumatoid arthritis, and systemic lupus erythematosus, have been shown to have increased clinical severity and/or relapse rates in the early spring, which are coincident with higher occurrence of MD in spring (40).

Overall, these comorbidities may help explain seasonal fluctuations in MD occurrence, but their specific roles in this seasonal pattern remain uncertain.

### 4.3 Exploratory subgroup analyses

Subgroup analyses according to geographic region were performed because the prevalence of MD has been reported to vary across ethnic and geographic populations (41). Although no significant subgroup differences were observed in the spring or winter comparisons, a significant subgroup difference was identified in the autumn comparison. However, because only one study was available from Europe, the reliability of regional comparisons was limited and the observed difference should be interpreted with caution. Further high-quality studies from diverse geographic regions are needed to clarify whether seasonal variation in MD differs across populations.

We also performed subgroup analyses according to data source, as different data sources may reflect different outcome types related to MD occurrence. Although no statistically significant subgroup difference was detected, the findings suggest that outcome type related to data source may influence the assessment of seasonal variation in MD. Administrative database studies mainly reflect insurance claims and healthcare utilization, whereas hospital-based studies may capture clinical visits or acute presentations. Therefore, differences in data source and outcome definition should be considered when interpreting the observed seasonal patterns.

### 4.4 Public health implications

To our knowledge, this study is the first comprehensive synthesis of evidence on seasonal variations in MD. Our findings suggest that the occurrence of MD may exhibit seasonal fluctuations, which may provide a new perspective for MD. However, the observed effect sizes were relatively modest, and a substantial proportion of the pooled data was derived from an administrative database study. Therefore, it remains unclear whether the observed seasonal variation reflects a true biological phenomenon or is partly influenced by healthcare utilization patterns and database-related factors. Consequently, the clinical significance of these findings should be interpreted with caution, and further prospective studies are required to validate the observed associations.

### 4.5 Limitations

However, this meta-analysis has several limitations. First, due to the limited number of available studies, we included studies with different designs and outcome types and collectively considered them as reflecting the occurrence of MD. Although subgroup analyses were performed to explore the potential impact of these differences, some degree of conceptual heterogeneity may remain. Second, the number of eligible studies was relatively small, which limited the assessment of publication bias and restricted the ability to perform more detailed subgroup analyses. Therefore, the results of the funnel plots and Egger’s test should be interpreted with caution. Third, the geographical distribution of the included studies is limited. Only one study is from Europe, while the rest are from Asia. Consequently, the applicability of the findings to other regions and ethnic groups remains uncertain. In addition, this study did not address confounding factors such as medical history, comorbidities, lifestyle, hospital practices, and healthcare accessibility. Because these factors were not consistently reported across studies, their potential influence on the observed seasonal variation in MD could not be fully assessed. Therefore, the potential influence of confounding factors on the observed results cannot be excluded. Prospective studies with large sample size employing standardized methodologies are needed to further investigate the relationship between MD and meteorological parameters.

## 5 Conclusion

Our meta-analysis results suggest that MD may exhibit seasonal variations, with slightly higher occurrence in spring and declining occurrence in winter. Nevertheless, the precise magnitude should be interpreted cautiously given the modest effect sizes and limited number of available reports. While the biological mechanisms underlying these seasonal associations remain unclear, future studies incorporating environmental, meteorological, and physiological variables are warranted to better understand the seasonal variation in MD.

## Data Availability

The original contributions presented in the study are included in the article/Supplementary material, further inquiries can be directed to the corresponding authors.
