# Supplementary material for: Seasonal variation in Ménière disease: a systematic review and meta-analysis
Source: Front Neurol. 2026 Jul 2;17:1876181. doi: 10.3389/fneur.2026.1876181 (PMC13372600; doi:10.3389/fneur.2026.1876181)
Supplement: Supplementary file 1 [file Supplementary_file_1.DOCX]

Supplementary Material 1: Search strategy

# PubMed

1 (((((((((((((((((((((((((((((((((("Meniere Disease"[Mesh]) OR (Disease, Meniere[Title/Abstract])) OR (Meniere's Disease[Title/Abstract])) OR (Disease, Meniere's[Title/Abstract])) OR (Menieres Disease[Title/Abstract])) OR (Meniere's Syndrome[Title/Abstract])) OR (Menieres Syndrome[Title/Abstract])) OR (Meniere Syndrome[Title/Abstract])) OR (Syndrome, Meniere's[Title/Abstract])) OR (Ménière Disease[Title/Abstract])) OR (Disease, Ménière[Title/Abstract])) OR (Diseases, Ménière[Title/Abstract])) OR (Ménière Diseases[Title/Abstract])) OR (Ménière's Disease[Title/Abstract])) OR (Disease, Ménière's[Title/Abstract])) OR (Diseases, Ménière's[Title/Abstract])) OR (Ménières Disease[Title/Abstract])) OR (Ménière's Diseases[Title/Abstract])) OR (Vertigo, Aural[Title/Abstract])) OR (Auditory Vertigo[Title/Abstract])) OR (Auditory Vertigos[Title/Abstract])) OR (Vertigo, Auditory[Title/Abstract])) OR (Vertigos, Auditory[Title/Abstract])) OR (Aural Vertigo[Title/Abstract])) OR (Otogenic Vertigo[Title/Abstract])) OR (Otogenic Vertigos[Title/Abstract])) OR (Vertigo, Otogenic[Title/Abstract])) OR (Vertigos, Otogenic[Title/Abstract])) OR (Ménière's Vertigo[Title/Abstract])) OR (Ménières Vertigo[Title/Abstract])) OR (Ménière's Vertigos[Title/Abstract])) OR (Ménière Vertigo[Title/Abstract])) OR (Vertigo, Ménière's[Title/Abstract])) OR (Vertigos, Ménière's[Title/Abstract]))

2 ("Endolymphatic Hydrops"[Mesh]) OR (Hydrops, Endolymphatic[Title/Abstract])

3 ((((((((((((((((((((((((((((((((((("Meniere Disease"[Mesh]) OR (Disease, Meniere[Title/Abstract])) OR (Meniere's Disease[Title/Abstract])) OR (Disease, Meniere's[Title/Abstract])) OR (Menieres Disease[Title/Abstract])) OR (Meniere's Syndrome[Title/Abstract])) OR (Menieres Syndrome[Title/Abstract])) OR (Meniere Syndrome[Title/Abstract])) OR (Syndrome, Meniere's[Title/Abstract])) OR (Ménière Disease[Title/Abstract])) OR (Disease, Ménière[Title/Abstract])) OR (Diseases, Ménière[Title/Abstract])) OR (Ménière Diseases[Title/Abstract])) OR (Ménière's Disease[Title/Abstract])) OR (Disease, Ménière's[Title/Abstract])) OR (Diseases, Ménière's[Title/Abstract])) OR (Ménières Disease[Title/Abstract])) OR (Ménière's Diseases[Title/Abstract])) OR (Vertigo, Aural[Title/Abstract])) OR (Auditory Vertigo[Title/Abstract])) OR (Auditory Vertigos[Title/Abstract])) OR (Vertigo, Auditory[Title/Abstract])) OR (Vertigos, Auditory[Title/Abstract])) OR (Aural Vertigo[Title/Abstract])) OR (Otogenic Vertigo[Title/Abstract])) OR (Otogenic Vertigos[Title/Abstract])) OR (Vertigo, Otogenic[Title/Abstract])) OR (Vertigos, Otogenic[Title/Abstract])) OR (Ménière's Vertigo[Title/Abstract])) OR (Ménières Vertigo[Title/Abstract])) OR (Ménière's Vertigos[Title/Abstract])) OR (Ménière Vertigo[Title/Abstract])) OR (Vertigo, Ménière's[Title/Abstract])) OR (Vertigos, Ménière's[Title/Abstract]))) OR (("Endolymphatic Hydrops"[Mesh]) OR (Hydrops, Endolymphatic[Title/Abstract]))

4 (((((("Seasons"[Mesh]) OR (Season[Title/Abstract])) OR (Seasonal Variation[Title/Abstract])) OR (Seasonal Variations[Title/Abstract])) OR (Variation, Seasonal[Title/Abstract])) OR (Variations, Seasonal[Title/Abstract]))

5 ("Climate"[Mesh]) OR (Climates[Title/Abstract])

6 (((("Air Pressure"[Mesh]) OR (Air Pressures[Title/Abstract])) OR (Pressure, Air[Title/Abstract])) OR (Pressures, Air[Title/Abstract]))

7 ("Humidity"[Mesh]) OR (Humidities[Title/Abstract])

8 ((("Weather"[Mesh]) OR (Fog[Title/Abstract])) OR (Fogs[Title/Abstract]))

9 "Air"[Mesh]

10 ((((((((((("Seasons"[Mesh]) OR (Season[Title/Abstract])) OR (Seasonal Variation[Title/Abstract])) OR (Seasonal Variations[Title/Abstract])) OR (Variation, Seasonal[Title/Abstract])) OR (Variations, Seasonal[Title/Abstract]))) OR (("Climate"[Mesh]) OR (Climates[Title/Abstract]))) OR ((((("Air Pressure"[Mesh]) OR (Air Pressures[Title/Abstract])) OR (Pressure, Air[Title/Abstract])) OR (Pressures, Air[Title/Abstract])))) OR (("Humidity"[Mesh]) OR (Humidities[Title/Abstract]))) OR (((("Weather"[Mesh]) OR (Fog[Title/Abstract])) OR (Fogs[Title/Abstract])))) OR ("Air"[Mesh])

11 (((((((((((((((((((((((((((((((((((("Meniere Disease"[Mesh]) OR (Disease, Meniere[Title/Abstract])) OR (Meniere's Disease[Title/Abstract])) OR (Disease, Meniere's[Title/Abstract])) OR (Menieres Disease[Title/Abstract])) OR (Meniere's Syndrome[Title/Abstract])) OR (Menieres Syndrome[Title/Abstract])) OR (Meniere Syndrome[Title/Abstract])) OR (Syndrome, Meniere's[Title/Abstract])) OR (Ménière Disease[Title/Abstract])) OR (Disease, Ménière[Title/Abstract])) OR (Diseases, Ménière[Title/Abstract])) OR (Ménière Diseases[Title/Abstract])) OR (Ménière's Disease[Title/Abstract])) OR (Disease, Ménière's[Title/Abstract])) OR (Diseases, Ménière's[Title/Abstract])) OR (Ménières Disease[Title/Abstract])) OR (Ménière's Diseases[Title/Abstract])) OR (Vertigo, Aural[Title/Abstract])) OR (Auditory Vertigo[Title/Abstract])) OR (Auditory Vertigos[Title/Abstract])) OR (Vertigo, Auditory[Title/Abstract])) OR (Vertigos, Auditory[Title/Abstract])) OR (Aural Vertigo[Title/Abstract])) OR (Otogenic Vertigo[Title/Abstract])) OR (Otogenic Vertigos[Title/Abstract])) OR (Vertigo, Otogenic[Title/Abstract])) OR (Vertigos, Otogenic[Title/Abstract])) OR (Ménière's Vertigo[Title/Abstract])) OR (Ménières Vertigo[Title/Abstract])) OR (Ménière's Vertigos[Title/Abstract])) OR (Ménière Vertigo[Title/Abstract])) OR (Vertigo, Ménière's[Title/Abstract])) OR (Vertigos, Ménière's[Title/Abstract]))) OR (("Endolymphatic Hydrops"[Mesh]) OR (Hydrops, Endolymphatic[Title/Abstract]))) AND (((((((((((("Seasons"[Mesh]) OR (Season[Title/Abstract])) OR (Seasonal Variation[Title/Abstract])) OR (Seasonal Variations[Title/Abstract])) OR (Variation, Seasonal[Title/Abstract])) OR (Variations, Seasonal[Title/Abstract]))) OR (("Climate"[Mesh]) OR (Climates[Title/Abstract]))) OR ((((("Air Pressure"[Mesh]) OR (Air Pressures[Title/Abstract])) OR (Pressure, Air[Title/Abstract])) OR (Pressures, Air[Title/Abstract])))) OR (("Humidity"[Mesh]) OR (Humidities[Title/Abstract]))) OR (((("Weather"[Mesh]) OR (Fog[Title/Abstract])) OR (Fogs[Title/Abstract])))) OR ("Air"[Mesh]))

# The Cochrane Library

#1 MeSH descriptor: [Meniere Disease] explode all trees

#2 (Disease, Meniere):ti,ab,kw OR (Meniere's Disease):ti,ab,kw OR (Disease, Meniere's):ti,ab,kw OR (Menieres Disease):ti,ab,kw OR (Meniere's Syndrome):ti,ab,kw OR (Menieres Syndrome):ti,ab,kw OR (Meniere Syndrome):ti,ab,kw OR (Syndrome, Meniere's):ti,ab,kw OR (Ménière Disease):ti,ab,kw OR (Disease, Ménière):ti,ab,kw OR (Diseases, Ménière):ti,ab,kw OR (Ménière Diseases):ti,ab,kw OR (Ménière's Disease):ti,ab,kw OR (Disease, Ménière's):ti,ab,kw OR (Diseases, Ménière's):ti,ab,kw OR (Ménières Disease):ti,ab,kw OR (Ménière's Diseases):ti,ab,kw OR (Vertigo, Aural):ti,ab,kw OR (Auditory Vertigo):ti,ab,kw OR (Auditory Vertigos):ti,ab,kw OR (Vertigo, Auditory):ti,ab,kw OR (Vertigos, Auditory):ti,ab,kw OR (Aural Vertigo):ti,ab,kw OR (Otogenic Vertigo):ti,ab,kw OR (Otogenic Vertigos):ti,ab,kw OR (Vertigo, Otogenic):ti,ab,kw OR (Vertigos, Otogenic):ti,ab,kw OR (Ménière's Vertigo):ti,ab,kw OR (Ménières Vertigo):ti,ab,kw OR (Ménière's Vertigos):ti,ab,kw OR (Ménière Vertigo):ti,ab,kw OR (Vertigo, Ménière's):ti,ab,kw OR (Vertigos, Ménière's):ti,ab,kw

#3 #1 OR #2

#4 MeSH descriptor: [Endolymphatic Hydrops] explode all trees

#5 (Hydrops, Endolymphatic):ti,ab,kw

#6 #4 OR #5

#7 #3 OR #6

#8 MeSH descriptor: [Seasons] explode all trees

#9 (Season):ti,ab,kw OR (Seasonal Variation):ti,ab,kw OR (Seasonal Variations):ti,ab,kw OR (Variation, Seasonal):ti,ab,kw OR (Variations, Seasonal):ti,ab,kw

#10 #8 OR #9

#11 MeSH descriptor: [Climate] explode all trees

#12 (Climates):ti,ab,kw

#13 #11 OR #12

#14 MeSH descriptor: [Air Pressure] explode all trees

#15 (Air Pressures):ti,ab,kw OR (Pressure, Air):ti,ab,kw OR (Pressures, Air):ti,ab,kw

#16 #14 OR #15

#17 MeSH descriptor: [Humidity] explode all trees

#18 (Humidities):ti,ab,kw

#19 #17 OR #18

#20 MeSH descriptor: [Weather] explode all trees

#21 (Fog):ti,ab,kw OR (Fogs):ti,ab,kw

#22 #20 OR #21

#23 MeSH descriptor: [Air] explode all trees

#24 #10 OR #13 OR #16 OR #19 OR #22 OR #23

#25 #7 AND #24

# Embase

| No. | Query | Results |
| --- | --- | --- |
| #21 | (('meniere disease'/exp OR 'meniere disease') OR ('meniere disease':ab,kw,ti OR 'disease, meniere':ab,kw,ti OR 'menieres disease':ab,kw,ti OR 'menieres syndrome':ab,kw,ti OR 'meniere syndrome':ab,kw,ti OR 'ménière disease':ab,kw,ti OR 'disease, ménière':ab,kw,ti OR 'diseases, ménière':ab,kw,ti OR 'ménière diseases':ab,kw,ti OR 'ménières disease':ab,kw,ti OR 'vertigo, aural':ab,kw,ti OR 'auditory vertigo':ab,kw,ti OR 'auditory vertigos':ab,kw,ti OR 'vertigo, auditory':ab,kw,ti OR 'vertigos, auditory':ab,kw,ti OR 'aural vertigo':ab,kw,ti OR 'otogenic vertigo':ab,kw,ti OR 'otogenic vertigos':ab,kw,ti OR 'vertigo, otogenic':ab,kw,ti OR 'vertigos, otogenic':ab,kw,ti OR 'ménières vertigo':ab,kw,ti OR 'ménière vertigo':ab,kw,ti OR 'endolymphatic hydrops':ab,kw,ti OR 'hydrops, endolymphatic':ab,kw,ti)) AND ((('season'/exp OR 'season') OR ('seasons':ab,kw,ti OR 'season':ab,kw,ti OR 'seasonal variation':ab,kw,ti OR 'seasonal variations':ab,kw,ti OR 'variation, seasonal':ab,kw,ti OR 'variations, seasonal':ab,kw,ti)) OR (('climate'/exp OR 'climate') OR ('climate':ab,kw,ti OR 'climates':ab,kw,ti)) OR (('air pressure'/exp OR 'air pressure') OR ('air pressure':ab,kw,ti OR 'air pressures':ab,kw,ti OR 'pressure, air':ab,kw,ti OR 'pressures, air':ab,kw,ti)) OR ('air'/exp OR 'air') OR (('humidity'/exp OR 'humidity') OR ('humidity':ab,kw,ti OR 'humidities':ab,kw,ti)) OR (('weather'/exp OR 'weather') OR ('weather':ab,kw,ti OR 'fog':ab,kw,ti OR 'fogs':ab,kw,ti))) | 263 |
| #20 | (('season'/exp OR 'season') OR ('seasons':ab,kw,ti OR 'season':ab,kw,ti OR 'seasonal variation':ab,kw,ti OR 'seasonal variations':ab,kw,ti OR 'variation, seasonal':ab,kw,ti OR 'variations, seasonal':ab,kw,ti)) OR (('climate'/exp OR 'climate') OR ('climate':ab,kw,ti OR 'climates':ab,kw,ti)) OR (('air pressure'/exp OR 'air pressure') OR ('air pressure':ab,kw,ti OR 'air pressures':ab,kw,ti OR 'pressure, air':ab,kw,ti OR 'pressures, air':ab,kw,ti)) OR ('air'/exp OR 'air') OR (('humidity'/exp OR 'humidity') OR ('humidity':ab,kw,ti OR 'humidities':ab,kw,ti)) OR (('weather'/exp OR 'weather') OR ('weather':ab,kw,ti OR 'fog':ab,kw,ti OR 'fogs':ab,kw,ti)) | 1228467 |
| #19 | ('weather'/exp OR 'weather') OR ('weather':ab,kw,ti OR 'fog':ab,kw,ti OR 'fogs':ab,kw,ti) | 107816 |
| #18 | ('humidity'/exp OR 'humidity') OR ('humidity':ab,kw,ti OR 'humidities':ab,kw,ti) | 121925 |
| #17 | 'air'/exp OR 'air' | 632522 |
| #16 | 'weather':ab,kw,ti OR 'fog':ab,kw,ti OR 'fogs':ab,kw,ti | 45392 |
| #15 | 'weather'/exp OR 'weather' | 100001 |
| #14 | 'humidity':ab,kw,ti OR 'humidities':ab,kw,ti | 57056 |
| #13 | 'humidity'/exp OR 'humidity' | 121569 |
| #12 | ('air pressure'/exp OR 'air pressure') OR ('air pressure':ab,kw,ti OR 'air pressures':ab,kw,ti OR 'pressure, air':ab,kw,ti OR 'pressures, air':ab,kw,ti) | 5406 |
| #11 | 'air pressure':ab,kw,ti OR 'air pressures':ab,kw,ti OR 'pressure, air':ab,kw,ti OR 'pressures, air':ab,kw,ti | 4505 |
| #10 | 'air pressure'/exp OR 'air pressure' | 4831 |
| #9 | ('climate'/exp OR 'climate') OR ('climate':ab,kw,ti OR 'climates':ab,kw,ti) | 242483 |
| #8 | 'climate':ab,kw,ti OR 'climates':ab,kw,ti | 165131 |
| #7 | 'climate'/exp OR 'climate' | 237856 |
| #6 | ('season'/exp OR 'season') OR ('seasons':ab,kw,ti OR 'season':ab,kw,ti OR 'seasonal variation':ab,kw,ti OR 'seasonal variations':ab,kw,ti OR 'variation, seasonal':ab,kw,ti OR 'variations, seasonal':ab,kw,ti) | 319918 |
| #5 | 'seasons':ab,kw,ti OR 'season':ab,kw,ti OR 'seasonal variation':ab,kw,ti OR 'seasonal variations':ab,kw,ti OR 'variation, seasonal':ab,kw,ti OR 'variations, seasonal':ab,kw,ti | 172711 |
| #4 | 'season'/exp OR 'season' | 301248 |
| #3 | ('meniere disease'/exp OR 'meniere disease') OR ('meniere disease':ab,kw,ti OR 'disease, meniere':ab,kw,ti OR 'menieres disease':ab,kw,ti OR 'menieres syndrome':ab,kw,ti OR 'meniere syndrome':ab,kw,ti OR 'ménière disease':ab,kw,ti OR 'disease, ménière':ab,kw,ti OR 'diseases, ménière':ab,kw,ti OR 'ménière diseases':ab,kw,ti OR 'ménières disease':ab,kw,ti OR 'vertigo, aural':ab,kw,ti OR 'auditory vertigo':ab,kw,ti OR 'auditory vertigos':ab,kw,ti OR 'vertigo, auditory':ab,kw,ti OR 'vertigos, auditory':ab,kw,ti OR 'aural vertigo':ab,kw,ti OR 'otogenic vertigo':ab,kw,ti OR 'otogenic vertigos':ab,kw,ti OR 'vertigo, otogenic':ab,kw,ti OR 'vertigos, otogenic':ab,kw,ti OR 'ménières vertigo':ab,kw,ti OR 'ménière vertigo':ab,kw,ti OR 'endolymphatic hydrops':ab,kw,ti OR 'hydrops, endolymphatic':ab,kw,ti) | 12431 |
| #2 | 'meniere disease':ab,kw,ti OR 'disease, meniere':ab,kw,ti OR 'menieres disease':ab,kw,ti OR 'menieres syndrome':ab,kw,ti OR 'meniere syndrome':ab,kw,ti OR 'ménière disease':ab,kw,ti OR 'disease, ménière':ab,kw,ti OR 'diseases, ménière':ab,kw,ti OR 'ménière diseases':ab,kw,ti OR 'ménières disease':ab,kw,ti OR 'vertigo, aural':ab,kw,ti OR 'auditory vertigo':ab,kw,ti OR 'auditory vertigos':ab,kw,ti OR 'vertigo, auditory':ab,kw,ti OR 'vertigos, auditory':ab,kw,ti OR 'aural vertigo':ab,kw,ti OR 'otogenic vertigo':ab,kw,ti OR 'otogenic vertigos':ab,kw,ti OR 'vertigo, otogenic':ab,kw,ti OR 'vertigos, otogenic':ab,kw,ti OR 'ménières vertigo':ab,kw,ti OR 'ménière vertigo':ab,kw,ti OR 'endolymphatic hydrops':ab,kw,ti OR 'hydrops, endolymphatic':ab,kw,ti | 3198 |
| #1 | 'meniere disease'/exp OR 'meniere disease' | 12079 |

# Web of Science

1 (((((((((((((((((((((((((((((((((((TS=(Meniere Disease)) OR TS=(Disease, Meniere)) OR TS=(Meniere's Disease)) OR TS=(Disease, Meniere's)) OR TS=(Menieres Disease)) OR TS=(Meniere's Syndrome)) OR TS=(Menieres Syndrome)) OR TS=(Meniere Syndrome)) OR TS=(Syndrome, Meniere's)) OR TS=(Ménière Disease)) OR TS=(Disease, Ménière)) OR TS=(Diseases, Ménière)) OR TS=(Ménière Diseases)) OR TS=(Ménière's Disease)) OR TS=(Disease, Ménière's)) OR TS=(Diseases, Ménière's)) OR TS=(Ménières Disease)) OR TS=(Ménière's Diseases)) OR TS=(Vertigo, Aural)) OR TS=(Auditory Vertigo)) OR TS=(Auditory Vertigos)) OR TS=(Vertigo, Auditory)) OR TS=(Vertigos, Auditory)) OR TS=(Aural Vertigo)) OR TS=(Otogenic Vertigo)) OR TS=(Otogenic Vertigos)) OR TS=(Vertigo, Otogenic)) OR TS=(Vertigos, Otogenic)) OR TS=(Ménière's Vertigo)) OR TS=(Ménières Vertigo)) OR TS=(Ménière's Vertigos)) OR TS=(Ménière Vertigo)) OR TS=(Vertigo, Ménière's)) OR TS=(Vertigos, Ménière's)) OR TS=( Endolymphatic Hydrops)) OR TS=(Hydrops, Endolymphatic)

2 (((((TS=(Seasons)) OR TS=(Season)) OR TS=(Seasonal Variation)) OR TS=(Seasonal Variations)) OR TS=(Variation, Seasonal)) OR TS=(Variations, Seasonal)

3 (TS=(Climate)) OR TS=(Climates)

4 (((TS=(Air Pressure)) OR TS=(Air Pressures)) OR TS=(Pressure, Air)) OR TS=(Pressures, Air)

5 (TS=(Humidity)) OR TS=(Humidities)

6 ((TS=(Weather)) OR TS=(Fog)) OR TS=(Fogs)

7 TS=(Air)

8 #7 OR #6 OR #5 OR #4 OR #3 OR #2

9: #8 AND #1

# SinoMed

(("气候"[常用字段:智能] OR "习服"[常用字段:智能] OR "气压"[常用字段:智能] OR "湿度"[常用字段:智能] OR "潮湿"[常用字段:智能] OR "天气"[常用字段:智能] OR "空气"[常用字段:智能]) OR ("季节"[常用字段:智能] OR "季节性"[常用字段:智能] OR "季节性变化"[常用字段:智能] OR "季节差异"[常用字段:智能] OR "季节性差异"[常用字段:智能] OR "季节变化特征"[常用字段:智能])) AND ("梅尼埃病"[常用字段:智能] OR "梅尼埃病眩晕"[常用字段:智能] OR "美尼尔综合征"[常用字段:智能] OR "美尼尔氏眩晕症"[常用字段:智能] OR "梅尼埃氏病"[常用字段:智能] OR "梅尼埃氏病"[常用字段:智能] OR "内淋巴积液"[常用字段:智能])

# Wanfang

主题:(梅尼埃病 OR 梅尼埃病眩晕 OR 美尼尔综合征 OR 美尼尔氏眩晕症 OR 梅尼埃氏病 OR 内淋巴积液) and 主题:(季节 OR 季节性 OR 季节性变化 OR 季节差异 OR 季节性差异 OR 季节变化特征 OR 气候 OR 习服 OR 气压 OR 湿度 OR 潮湿 OR 天气 OR 空气)

# CNKI

（主题：季节 + 季节性 + 季节性变化 + 季节差异 + 季节性差异 + 季节变化特征）OR（主题：气候 + 气候因子 + 气候影响 + 气候因素 + 习服）OR（主题：气压 + 气压变化 + 不同气压）OR（主题：湿度 + 湿度影响 + 温度和湿度 + 空气湿度）OR（主题：天气 + 天气条件 + 天气影响 + 天气学）OR（主题：空气 + 空气湿度 + 空气温度 + 环境空气）AND（主题：梅尼埃病 + 梅尼埃病眩晕 + 美尼尔综合征 + 美尼尔氏眩晕症 + 梅尼埃氏病 + 内淋巴积液）
